# PIN7 Auxin Carrier Has a Preferential Role in Terminating Radial Root Expansion in *Arabidopsis thaliana*

**DOI:** 10.3390/ijms19041238

**Published:** 2018-04-19

**Authors:** Michel Ruiz Rosquete, Sascha Waidmann, Jürgen Kleine-Vehn

**Affiliations:** Department of Applied Genetics and Cell Biology, University of Natural Resources and Life Sciences (BOKU), Vienna 1190, Austria; michelrr1975@yahoo.es (M.R.R.); sascha.waidmann@boku.ac.at (S.W.)

**Keywords:** lateral root, gravitropism, auxin, PIN4, PIN3, PIN7, gravitropic set point angle, GSA, root system architecture

## Abstract

Directional growth of lateral roots is critical for radial expansion and soil coverage. Despite its importance, almost nothing is known about its molecular determinants. Initially, young lateral roots (LRs) grow away from the parental root, maintaining the angle acquired shortly after emergence. A second downwards bending response to gravity terminates the so-called plateau phase and thereby limits radial root expansion. Here, we show that the exit from the plateau phase correlates with an increase in auxin signalling at the tip of the LRs. Moreover, the increase in auxin levels induces the termination of the plateau phase, which requires PIN-FORMED (PIN) auxin efflux carriers. Our data suggests that the developmental increase in auxin triggers the preferential derepression of PIN7 in gravity-sensing columella cells. The subsequent polarization of PIN7 heralds the bending towards gravity and, hence, the exit from the plateau phase. This developmental framework reveals the distinct roles of PIN auxin efflux carriers in controlling the radial growth of root systems.

## 1. Introduction

Secondary roots comprise the major biomass of mature root systems and, hence, their growth and orientation exert large influences on the overall plant performance. Lateral roots (LRs) differ from primary roots in their orientation with respect to the gravity vector, captured by the gravitropic set point angle (GSA) [1]. Initially, LRs grow away from the main axis of the primary root at non-vertical orientations, thus facilitating accessibility to the soil for water and nutrient acquisition and increasing mechanical support [2].

In roots, gravity is perceived in the columella cells of the root cap, but the actual growth response to gravity is achieved in the elongation zone. The growth regulator, auxin, provides the mobile link between the two parts [3,4,5,6]. This is achieved, in large part, thanks to the activity and polarized expression of several auxin transporters of the PIN-FORMED (PIN) family. In the main root, polar/asymmetric localization of PIN3, PIN4 and PIN7 in columella cells aligns the auxin transport towards gravity, which ultimately increases auxin levels at the lower root flank, inducing differential root growth and bending towards gravity [6].

**Figure 1:**
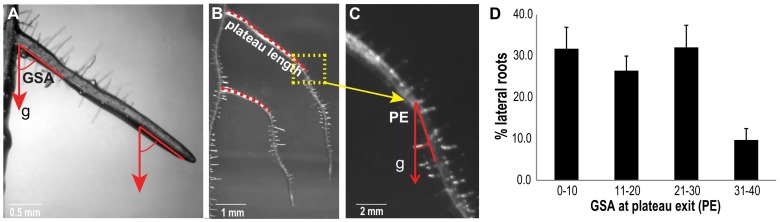
The gravitropic response at the plateau exit is highly variable. (**A**) During the plateau phase, or stage III, lateral roots (LRs) grow away from the parental root while keeping the initial (acquired in stage II) gravitropic set-point angle (GSA). Arrows denote the gravity (g) vector. (**B**) Two representative stage IV LRs are shown. Discontinuous red lines mark the length of their plateau trajectories. (**C**) Enlargement of the transit from stage III to IV, for the upper LR in B. (**D**) PE (GSA at plateau exit) distribution of Col-0 (wild type) LRs. Error bars represent SEM (*n* = 6 experiments; each experiment with *n* ≥ 20 seedlings (40–60 LRs)).

Young, stage I lateral roots emerge from the main root at a 90° angle, subsequently responding to gravity in stage II. This downwards bending is highly controlled, establishing an initial gravitropic set-point angle (GSA) of approximately 62° (Figure 1A) [7]. In this early phase of lateral root development, only PIN3 is expressed in the LR columella cells, where it establishes relatively weak asymmetric auxin signalling [7,8], which may, in part, explain the partial response to gravity of the LRs [7]. After this first gravitropic response, PIN3 expression diminishes and the LRs continue to grow, maintaining the acquired GSA [7]. We previously coined the term “plateau phase” for developmental stage III of the LRs, which is terminated by another gravitropic response (stage IV LRs) [7].

The initial establishment of the GSA and the length of the plateau phase determine the radial expansion of the root system. It currently remains unknown how this plateau-like growth phase of LRs is terminated. Our data suggest that a presumably auxin-dependent derepression of PIN auxin carriers in LR columella cells initiates the transition from stage III to stage IV LRs.

## 2. Results

### 2.1. The Plateau Length of Lateral Roots Contributes to Root System Architecture

The exit from the plateau phase is characterized by a second, positive gravitropic response, lowering the GSA to a value typically below 30° in stage IV LRs (Figure 1B–D). The GSA at the plateau exit (PE) fluctuated within a broad range (0–30°) (Figure 1C,D), suggesting that this developmental response is highly variable compared to the establishment of the initial GSA at stage II [7]. The initial GSA, the length of the plateau phase and its corresponding PE angle jointly determine the radial expansion of the root system.

**Figure 2:**
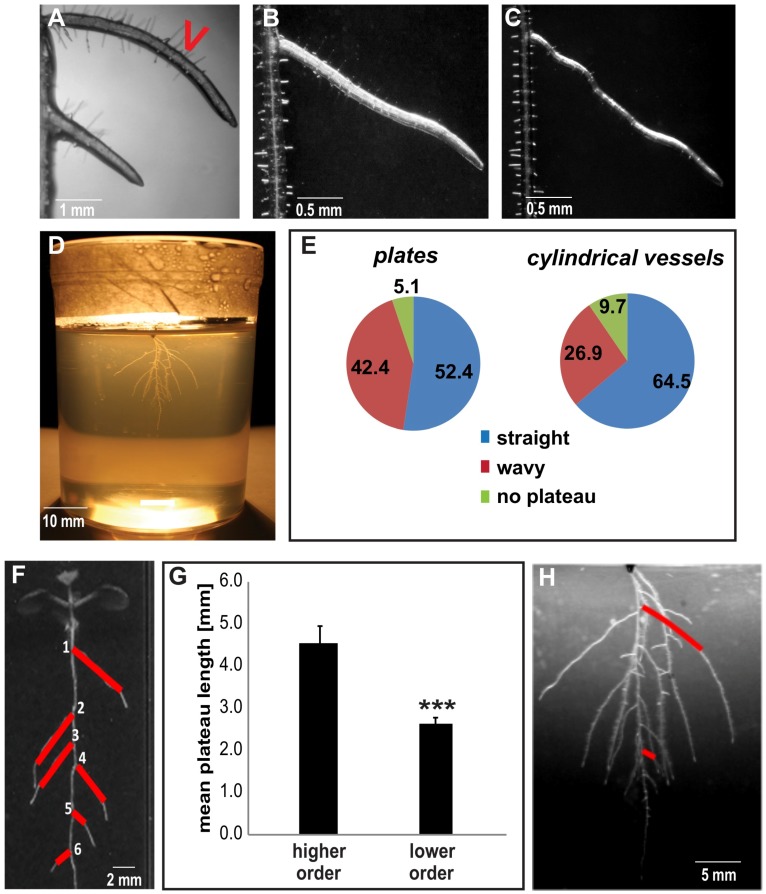
Characterization of the plateau phase. (**A**) A low percentage of LRs displayed a continuous decline of GSA throughout development (“no plateau”); notice the contrast between the upper LR (no plateau) and the bottom LR (in plateau phase). (**B**,**C**) Growth during the plateau phase may show some degree of waviness; compare with “straight” LR growth in (**A**) (bottom LR). The degree of waviness during plateau varies (LR depicted in (**C**) shows more waviness than LR depicted in (**B**). (**D**) A representative 20-DAG (days after germination) wild type seedling (Col-0), grown in a cylindrical vessel (3D model). (**E**) The incidence of waviness during plateau is lower in seedlings that grow within the agar (cylindrical vessels), compared to seedlings grown on the surface of a medium (plates). Percentages from a representative experiment are given (≥150 LRs; ≥30 seedlings). (**F**,**G**) Plateau segments (red lines in (**F**) of higher order LRs (1–3) are typically longer than those of younger or lower order LRs (4–6 in the example). Average values of plateau length were obtained for higher (1–3) and lower order LRs (4-) for individual Col-0 seedlings (**G**). Representative experiments are shown. Error bars represent SEM (*n* > 30 seedlings per experiment). *** *p* < 0.001 (Student’s *t*-test). A Representative 14-DAG Col-0 seedling is shown in (**F**). (**H**) Higher order LRs also display longer plateau (red lines) in the 3D growth model. A magnification of the seedling in (**D**) is shown. Red lines depict the plateau length.

Given the importance of the plateau length for root system architecture, we focus here on its regulation. Most individual LRs displayed a clearly distinguishable plateau phase; however, we also observed individual laterals that did not display a clear plateau but rather, showed a continuous decline in GSA throughout development (Figure 2A, upper LR). Although their frequency was rather low (5–10%) (Figure 2E), it remains to be seen whether this phenomenon reflects an alternative developmental program or whether it is due to some substrate constraint. On the other hand, wavy growth without major changes in the overall growth direction (GSA) is often observed during the plateau phase (Figure 2B,C). Waviness in *Arabidopsis* roots has been suggested to result from the mechanical torsion of the root at the 2D agar surface [9]. In agreement with this, we found that the frequency of “wavy” lateral roots diminished by approximately 40% when seedlings were grown within the gel of cylindrical vessels (3D in vitro system) (Figure 2D,E).

When observing the lateral roots along the main roots, we also noticed that positional effects may determine individual growth behaviour. The developmentally oldest LRs (more proximal to the hypocotyl) had longer plateau segments compared to those that emerged later on during root development (Figure 2F). Under our growth conditions, a plateau of the first three uppermost located laterals typically extended for 4–5 mm and their length were substantially longer compared to more distant, younger LRs (Figure 2F,G). Notably, we observed a similar effect in the 3D in vitro system (Figure 2H). We concluded that the first emerging LRs are most critical for the overall radial expansion of the root system in *Arabidopsis* (Figure 2F–H). Accordingly, we specifically investigated the plateau length of the first three LRs, which we refer to as “higher order lateral roots”.

### 2.2. Auxin Restricts Radial Expansion of Lateral Roots

**Figure 3:**
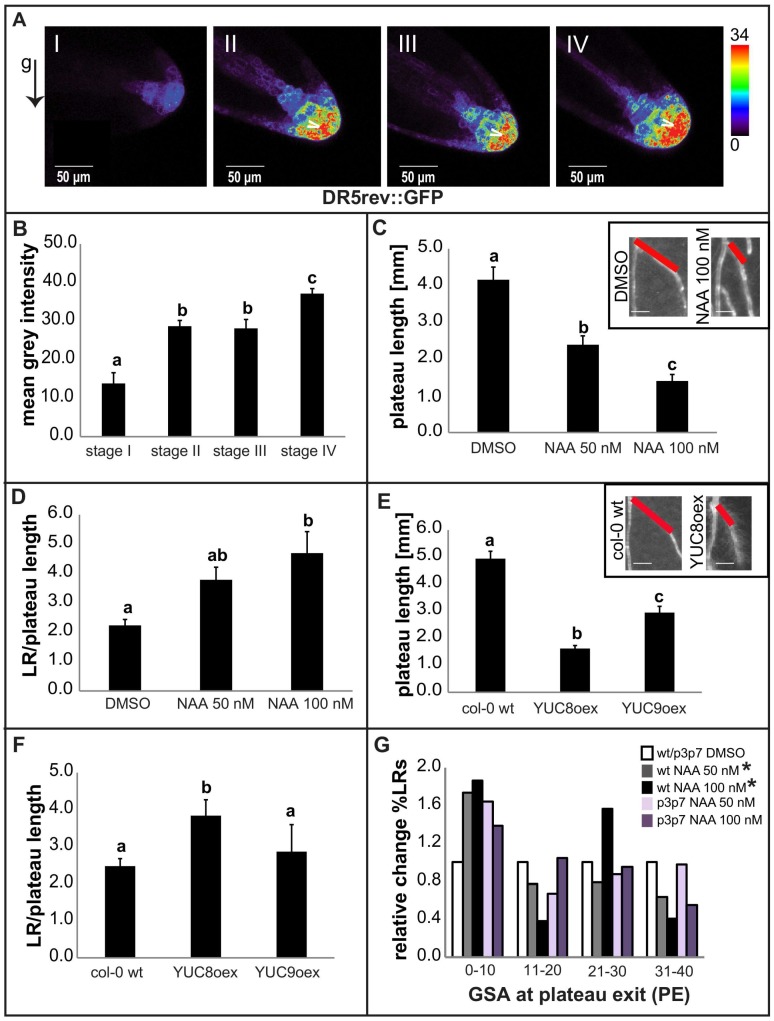
Auxin induces termination of the plateau phase. (**A**,**B**) Increases in auxin signaling at the root tip, visualized by DR5 (Direct Repeat 5) promoter-driven GFP (Green Fluorescent Protein) signal (**A**), accompany the transitions from stage I to II and from stage III to IV during LR development. Arrowheads denote areas of high DR5 activity. A semiquantitative color-coded heatmap of the DR5::GFP signal is provided. An arrow denotes the gravity (g) vector. (**B**) The mean intensity of the DR5::GFP reporter signal was measured in confocal microscopy images of the quiescent center + the columella region. A representative experiment is shown. Error bars represent SEM (*n* ≥ 10 LRs per stage, per experiment). (**C**,**D**) Treatments of 4-DAG Col-0 wild type seedlings with 50 and 100 nM auxin significantly reduced the plateau length (**C**) and increased the LR length/plateau length ratio (**D**). (**E**,**F**) Lines overexpressing the auxin biosynthetic genes, *YUC8* and *YUC9* (*YUCoex*), phenocopied the 1-Naphtaleneacetic acid (NAA) treatments. Insets in (**C**,**E**) illustrate how exogenous (**C**) and endogenous (**E**) increases in auxin cause a reduction in plateau length and a steeper plateau exit angle. Scale bars, 1 mm. A representative experiment is shown for both NAA treatments and *YUCoex* phenotyping. Error bars represent SEM (*n* > 30 LRs per experiment). The statistical significance in (**A**–**F**) was evaluated by one-way ANOVA followed by Tukey’s multiple comparison test. Distinct letters (a, b, c) indicate statistically significant differences (*p* < 0.05). (**G**) Auxin treatments caused a stronger graviresponse in the LRs of wild type (wt) seedlings, evidenced by the relative increase (percentage) in LRs exiting plateau at angles in the lowest category (0–10) of Plateau Exit (PE) distribution. *pin3 pin7* double mutants showed slight resistance to the auxin-determined shift in PE distribution. A representative experiment is shown (*n* > 30 LRs per experiment). Error bars represent SEM. * *p* < 0.05 (Kolmogorov–Smirnov test).

Lateral roots display age-dependent intensification of auxin signalling at their tips [10]. However, a possible correlation between increases in auxin signalling and transitions between LR developmental stages has not been explored. To test that possibility, we used the synthetic auxin responsive DR5 (Direct Repeat 5) promoter, driving a GFP (Green Fluorescent Protein), to quantify the levels of auxin signalling in the columella region of LRs in all four developmental stages. DR5::GFP activity was significantly augmented from stage I to II, correlating with the onset of auxin-dependent bending towards gravity (Figure 3A,B) [7]. Moreover, we observed a second significant increase in nuclear auxin signalling, which accompanied the transition from stage III to stage IV (Figure 3A,B).

Based on these observations, we assumed that an increase in auxin signalling could also play a role in regulating the second bending response to gravity, which terminates the plateau phase. To gain further evidence, we exogenously applied synthetic auxin 1-Naphtaleneacetic acid (NAA) in the nanomolar range, which strongly reduced the length of the plateau (Figure 3C). Overall lateral root growth was also reduced in those experiments; however, the reduction in the plateau length was comparatively greater (Figure 3D), suggesting that auxin negatively impacts the radial expansion of root systems by terminating the plateau phase. In agreement, the induction of YUCCA (YUC)-dependent auxin biosynthesis largely phenocopied the effect of auxin application, similarly reducing the plateau length of LRs (Figure 3E,F). Notably, auxin also caused a considerably increased frequency of laterals exiting the plateau at steeper angles (between 0° and 10°) (Figure 3G), reflecting a stronger response to gravity. This data suggests that both exogenous application and endogenous increase of auxin levels negatively limit the plateau length of LRs by stimulating their response to gravity.

### 2.3. Auxin Preferentially Triggers PIN7 Expression in Higher Order Lateral Roots

**Figure 4:**
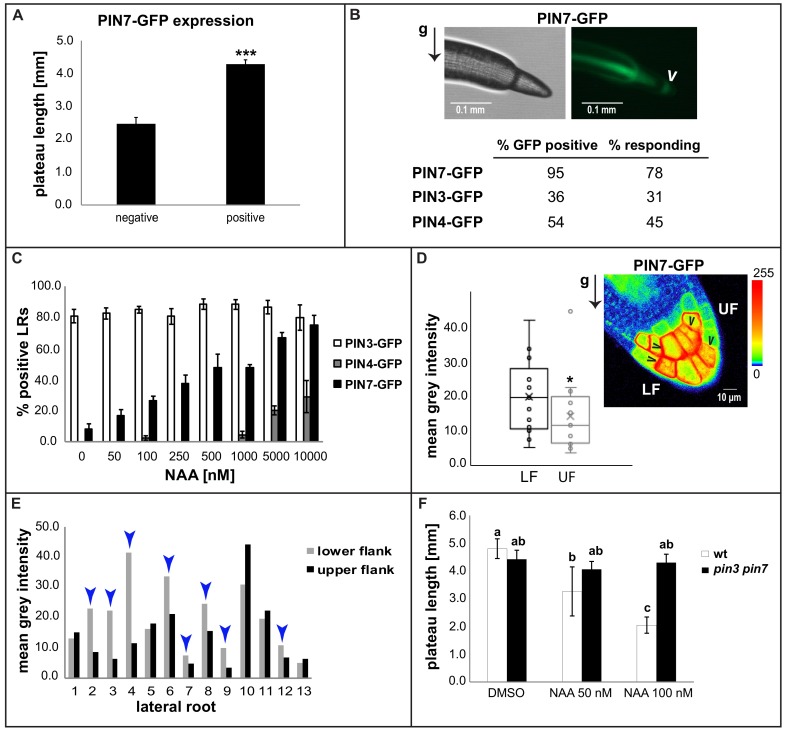
PIN7 has a preferential role in plateau phase termination. (**A**) Examination of higher order LRs (6–8 DAG) revealed a positive correlation between the likelihood of PIN7 expression and the length of the plateau phase. A representative experiment is shown (*n* ≥ 60 LRs; *n* ≥ 30 seedlings per experiment). *** *p* < 0.001 (Student’s *t*-test). (**B**) Compared with PIN3 and PIN4, PIN7 was more frequently detected (95%) in advanced stage III LRs (>4 mm). In 78% of tracked cases, the expression of PIN7-GFP at that developmental point correlated with the subsequent graviresponse (within 12 h), leading to exit from the plateau. An arrowhead indicates pPIN7-GFP expression at the root tip. An arrow denotes the gravity (g) vector. (**C**) 4-DAG seedlings of pPIN3::PIN3-GFP, pPIN4::PIN4-GFP and pPIN7::PIN7-GFP reporter lines were treated for three days with the indicated concentrations of NAA and the presence of a detectable signal of the corresponding PIN in the tip of stage I and II LRs was examined. An average of three experiments is shown (*n* ≥ 30 LRs per experiment). (**D**,**E**) The expression of PIN7-GFP in columella cells of the root tip exhibits polarization in a high percentage of late stage III LRs. Polarization was assessed by measurement of PIN7-GFP fluorescence intensity at lateral plasma membrane domains of the outermost columella cells at the lower flanks (LF) compared with those at the upper flanks (UF). Arrowheads indicate asymmetric PIN7-GFP distribution. A semiquantitative color-coded heat-map of the PIN7-GFP signal is provided (**D**). Results from a representative experiment are presented (*n* > 10 LRs, *n* > 7 seedlings per experiment) in (**D**,**E**). (**E**) displays the values of the individual LRs. The blue arrowheads in (**E**) indicate cases of polarization towards the gravity vector (LF > UF). One asterisk in D denotes *p* < 0.05 (Student’s *t*-test). (**F**) *pin3 pin7* mutants show resistance to the reduction of the plateau length caused by NAA treatments on the wild types (4-DAG seedlings). A representative experiment is shown (*n* > 30 LRs per experiment). Error bars represent SEM. Statistical significance was evaluated by two-way ANOVA followed by Tukey’s multiple comparison test. Distinct letters (a, b, c) indicate statistically significant differences (*p* < 0.05).

Next, we assessed the mechanism by which auxin affects the gravity responsiveness of plateau lateral roots. The expression of PIN auxin carriers in the columella cells of the root tip is crucial for the root gravitropic response [6]. One of the most intriguing characteristics of the plateau phase is the very low expression levels or even absence of all three columella PINs (PIN3, PIN4 and PIN7) at the transition from stage II to stage III LRs [7]. To assess whether the derepression of PINs heralds the exit from the plateau phase, we examined the expression of pPIN3::PIN3-GFP, pPIN4::PIN4-GFP and pPIN7::PIN7-GFP in higher order lateral roots. Even though the expression of the three PINs remained low during the plateau phase, we observed a tendency towards preferential PIN7-GFP derepression in older and hence, longer (≥4 mm) stage III lateral roots (Figure 4A). This prompted us to further explore the correlation between columella PIN expression and its requirement to exit from the plateau phase. Therefore, we inspected higher order LRs presumably facing the termination of their plateau phase (≥4 mm) (Figure 4B). PIN7 expression was frequent (95%) at the tip of those laterals, whereas expression of PIN3 and PIN4 occurred at much lower frequencies (36% and 54%, respectively). Auxin is known to feed back to PIN expression [11] and auxin signalling increased during lateral root maturation (Figure 3A,B). Therefore, we wondered whether auxin may impact distinctly on PIN7 expression in LRs. To assess this, we performed NAA treatments on stage I and stage II LRs, where PIN4 and PIN7 are strongly repressed [7]. The percentage of laterals expressing PIN4-GFP started to increase only at a very high concentration (1 μM), whereas PIN7-GFP showed steady induction starting at relatively low concentrations (50 nM) (Figure 4C). Hence, we conclude that PIN7 expression in LR columella cells is particularly sensitive to auxin.

### 2.4. Asymmetric PIN7-GFP Expression Correlates with Differential Growth Process

We next assessed whether PIN derepression in columella cells could indeed mediate stage transition and, hence, alterations in the growth direction of lateral roots. We accordingly tracked individual laterals with PIN-GFP expression, using a fluorescence binocular microscope, and recorded whether they actually altered their growth direction towards gravity within 12 h (Figure 4B). pPIN3::PIN3-GFP and pPIN4::PIN4-GFP positive stage III LRs displayed a plateau exit in 31% and 45% of the cases, respectively. This data shows that the majority of *PIN3* and *PIN4* expressing LRs did not respond to gravity, suggesting that neither *PIN3* nor *PIN4* expression in columella cells, per se, initiate gravitropic bending in late stage III LRs. In contrast, pPIN7::PIN7-GFP expression showed a rather high correlation (78%) with alterations in growth direction. This corroborates a preferential role of PIN7 during termination of the plateau phase, but also raises the question of why some PIN-GFP positive lateral roots do not display a differential growth response.

As PIN7-GFP was most readily expressed in higher order lateral roots and showed the highest correlation with alterations in growth behaviour, we subsequently analysed its subcellular localization, using confocal microscopy. We detected polarization of PIN7-GFP expression in only about 67% of the PIN7-GFP expressing LRs (Figure 4D,E). This is very much in range with the roughly 78% of PIN7-GFP positive lateral roots exiting the plateau phase. Accordingly, we conclude that not PIN7 expression as such, but rather its asymmetric pattern, largely correlates with differential growth in LR, transiting from stage III to IV. This finding also suggests that auxin-dependent derepression of mainly PIN7 terminates the radial expansion of the root system.

We observed an endogenous increase in auxin signalling at the transition from stage III to stage IV. Moreover, PIN7 expression showed relatively high auxin sensitivity. Accordingly, we hypothesize that auxin could be the actual developmental trigger to preferentially control PIN7 (and to a lesser extent, PIN3 and PIN4) derepression in stage III higher order LRs. This auxin-dependent regulation presumably initiates a PIN-dependent stage transition, reducing radial exploitation of the root system. Despite this presumed role for PIN7, both *pin7* as well as *pin3* single mutants remained sensitive to the auxin-induced shortening of the plateau phase (Supplementary Figure S1). The loss of a single *PIN* gene is compensated by ectopic upregulation of another redundant *PIN* gene [7,11] , which may mask the possibly distinct developmental role of single *PIN* genes in LRs [7]. In line with this anticipated functional redundancy, *pin3 pin7* double mutants were less sensitive to the auxin-induced reduction of the plateau length and the associated shift in PE (Figure 3G and Figure 4F). We therefore conclude that developmentally-defined auxin levels determine columella PIN expression in lateral roots, which is, in conjunction with a polarization mechanism, important for the auxin-dependent termination of radial lateral root growth.

## 3. Discussion

Three decades ago, Alistair Fitter noted that “many roots show a change from a diageotropic (horizontal with respect to the gravity vector) to a positively geotropic response after a period of growth, which results in an increase in the volume of soil exploited” [12]. By “after a period of growth”, Fitter most likely referred to what we call the plateau phase, an LR developmental stage of relevance for the initial spread of the root system. The molecular mechanisms and players that control the spatial expansion of root systems remain largely unexplored. With the present work, we have shed light on how the non-gravitropic growth during the plateau phase is terminated. We have shown that auxin plays a central role in the restriction of the plateau phase.

For auxin to steer root gravitropic responses, the activity and polar localization of auxin transporters at the plasma membrane are necessary [3,4,5,6,7,8]. In a previous work, we demonstrated a role for the auxin effluxer, PIN3, in the initial gravitropic response of young lateral roots [7]. Our new results propose an extended role for auxin as a negative regulator of the radial expansion of the root system. PIN-dependent auxin transport is similarly required to implement the second response to gravity. The data presented suggest that a different member of the PIN protein family, PIN7, mainly mediates the gravitropic response that determines the exit from the plateau phase. Notably, a developmentally defined increase in auxin signalling in the tip of LRs correlates with the transition from stage III to stage IV. We found that exogenously applied auxin preferentially induces the derepression of PIN7 in young LRs. Based on this data, it is very likely that the developmental increase in auxin signalling preferentially triggers PIN7 expression. In agreement, PIN7 is, compared to PIN3 and PIN4, more readily expressed at the tip of late stage III LRs. Moreover, PIN7 expression and subsequent polarization correlate most strongly with the gravitropic response. This set of data proposes a central role of PIN7 in determining the exit of LRs from the plateau phase. However, we do not rule out that PIN3 and PIN4 may also contribute to this developmental trait.

Currently, little is known about transcription factors that may differentially impact on columella PIN expression in LRs. Notably, the transcription factors MYB DOMAIN PROTEIN 88 (MYB88) and FOUR LIPS have been suggested to regulate both PIN3 and PIN7 expression in LRs [13]. It accordingly remains to be seen how the preferential and developmentally defined expression of PIN7 is molecularly achieved.

On the other hand, our data also shows that PIN3, PIN4, or PIN7 expression is not sufficient to induce differential growth and that additional polarity cues are required. AGCVIII kinases of the D6 and PINOID families impact on PIN phosphorylation and are known to modulate the localisation and/or transport activity of PINs at the plasma membrane [14,15,16,17]. However, very little is known about the phosphorylation of PIN7 and its potential contribution to LR growth. Intriguingly, *LAZY/DEEPER ROOTING* (*DRO*) genes were recently found to regulate columella PIN polarization towards gravity in main and lateral roots [18,19,20]. *DRO1* and *DRO2* show variable, age-dependent expression in LRs of *Arabidopsis* [20], which may differentially impact on PIN polarization in columella cells. Accordingly, it would be interesting to investigate whether *LAZY*/*DRO* genes indeed show stage dependent regulation in LRs.

In conclusion, we have substantiated our previous findings [7] and illustrated the preferential roles of PIN3 and PIN7 in early (stage I to II) and late (stage III to IV) gravity responses of LRs, respectively.

## 4. Materials and Methods

### 4.1. Plant Material and Growth Conditions

*Arabidopsis thaliana* (L.) Heyhn. seedlings of Columbia ecotype (Col-0) were used in this study. The following lines have been previously described: DR5::GFP [21], PIN3::PIN3-GFP [22], PIN7::PIN7-GFP, PIN4::PIN4-GFP [11], *pin7-1* [21], *pin3-5* [23], *pin3-5 pin7-1* [23], 35S::YUC8 [24] and 35S::YUC9 [24]. Seeds were sterilized with ethanol, plated on half Murashige and Skoog medium (1/2 MS salts (Duchefa Biochemie, Haarlem, The Netherlands), pH 5.9, 1% sucrose, and 0.8% agar), stratified at 4 °C for 2 days in the dark, and then transferred to a plant growth room (20–22 °C, 16 h light/8 h dark, vertical orientation). The same medium and growth conditions were used for seedlings grown in 68 × 100 mm cylindrical vessels (Greiner Bio-One, Kremsmünster, Austria), except for the use of a transparent Gelrite (Duchefa Biochemie, Haarlem, The Netherlands) instead of traditional plant agar.

### 4.2. Chemicals and Treatments

Treatments with 1-Naphtaleneacetic acid (NAA) (Duchefa Biochemie, Haarlem, The Netherlands) were performed on 4-day-old seedlings (transferred to supplemented media). NAA was dissolved in dimethylsulfoxide (DMSO) before addition to MS+ medium. Confocal and fluorescence microscope imaging were performed 3 days after treatment. Scanning of plates for plateau length/PE angle measurements was performed 8–10 days after treatments. All experiments were performed at least three times.

### 4.3. Microscopy

To detect PIN3-GFP, PIN4-GFP and PIN7-GFP signals in the tip of individual LRs, a binocular fluorescence microscope (Leica MZ FLIII; Leica Microsystems, Wetzlar, Germany) was used (GFP2 filters set: excitation filter 480/40 nm, barrier filter 510 nm) in combination with a DC 500 camera (Leica Microsystems, Wetzlar, Germany). To detect DR5::GFP and assess the asymmetry of PIN7-GFP signals, confocal microscopy was performed using a Leica DM6000 CS, 347 TCS AOBS (Leica Microsystems, Wetzlar, Germany) confocal laser scanning microscope, equipped with a HCX PL APO CS 348 20.0 0.70 IMM UV objective (Leica Microsystems, Wetzlar, Germany); excitation 488 nm, emission peak 509 nm). GFP signals in confocal images were analyzed and quantified using the software LAS AF 3.1 (Leica) and ImageJ (http://rsb.info.nih.gov/ij/; version 1.45j, NIH, Bethesda, MD, USA). All experiments were performed at least three times.

### 4.4. Measurements

Compared to our previous report on mainly stage II LRs [7], we particularly focused here on the stage III to IV transition. Therefore, we increased the concentration range of auxin, increased the sample numbers and assessed distinct parameters (see also figure legends). Plates with 12–14 DAG (days after germination) seedlings were scanned, and the plateau lengths and plateau exit (PE) angles of individual LRs were measured using the line and angle tools of ImageJ, respectively. PE angles were measured using the head of the gravity vector as a reference. GSA values at PE were sorted into 4 categories: 0–10°, 11–20°, 21–30°, and 31–40°. Percentages of incidence were calculated to generate graphs of PE distribution. Values for ratio LR/plateau length were obtained by dividing the total length of the lateral root by the length of its plateau phase. The mean intensity of auxin signalling in the tip of LRs, as reported by the marker line DR5::GFP, was quantified using the polygon tool of ImageJ, circumscribing an area comprising the columella and lateral root cap cells, up to the height of the quiescent centre. For the assessment of PIN7-GFP asymmetry across the root tip, the mean intensity of the GFP signal was estimated for lateral plasma membrane domains of individual columella cells facing the upper and lower sections, or flanks, of the lateral root cap. Values derived from the same section (LF and UF in Figure 3D,E) were averaged for each individual LR analyzed. “Polarization towards gravity” was defined when the average mean intensity PIN7-GFP values for membrane domains in the lower flank (LF) were bigger than the average mean intensity PIN7-GFP values for membrane domains in the upper flank (UF). All experiments were performed at least three times.

### 4.5. Statistics

The Kolmogorov–Smirnov test (*KS*-test) (http://www.physics.csbsju.edu/stats/KS-test.n.plot_form.html) was used to evaluate the statistical significance of differences observed between PE distributions. Student’s *t*-tests were used for two-group comparisons. ANOVA, followed by Tukey’s test, was used in the case of multiple comparisons to statistically evaluate differences between data sets. Information regarding sample sizes and usage of tests are depicted in figure legends.

## Supplementary Materials

Supplementary materials can be found at http://www.mdpi.com/1422-0067/19/4/1238/s1.
