# Supplementary material for: PIN7 Auxin Carrier Has a Preferential Role in Terminating Radial Root Expansion in Arabidopsis thaliana
Source: Int J Mol Sci. 2018 Apr 19;19(4):1238. doi: 10.3390/ijms19041238 (PMC5979548; doi:10.3390/ijms19041238)
Supplement: Supplementary file 1 [file ijms-19-01238-s001.pdf]

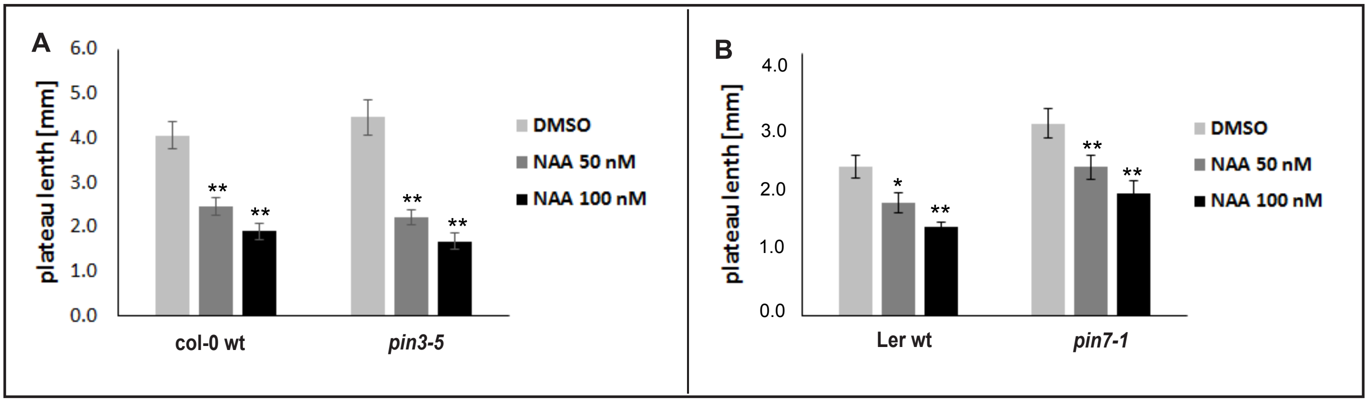

Supplemental Figure 1. Effect of auxin treatments on plateau length of *PIN3* and *PIN7* single mutants. A, B) 4-DAG seedlings of *pin3-5* (A) (in Col-0 wt background) and *pin7-1* (in Ler background) (B) remain responsive to the effect of auxin treatments on plateau length. A representative experiment is shown, for each mutant line and corresponding wt control. Error bars represent SEM ($n>30$ LRs per experiment). Statistical significance was evaluated by two-way ANOVA followed by multiple comparisons Tukey test. One ($p<0.05$) and two asterisks ($p<0.01$) denote statistically significant differences between NAA treatments and corresponding DMSO controls.
